# Anesthetic Isoflurane Posttreatment Attenuates Experimental Lung Injury by Inhibiting Inflammation and Apoptosis

**DOI:** 10.1155/2013/108928

**Published:** 2013-04-17

**Authors:** Jun-tang Li, Hui Wang, Wei Li, Li-feng Wang, Li-chao Hou, Jing-lan Mu, Xin Liu, Hui-juan Chen, Ke-lang Xie, Nan-lin Li, Chun-fang Gao

**Affiliations:** ^1^Institute of Anal-Colorectal Surgery, No. 150 Central Hospital of PLA, Luoyang, Henan 451000, China; ^2^Department of Immunology, State Key Laboratory of Cancer Biology, Fourth Military Medical University, Xi'an, Shaanxi 710032, China; ^3^Department of Vascular and Endocrine Surgery, Xijing Hospital, Fourth Military Medical University, Xi'an, Shaanxi 710032, China; ^4^Department of Biochemistry and Molecular Biology, State Key Laboratory of Cancer Biology, Fourth Military Medical University, Xi'an, Shaanxi 710032, China; ^5^Department of Anesthesiology, Xijing Hospital, Fourth Military Medical University, Xi'an, Shaanxi 710032, China; ^6^Department of Anesthesiology, General Hospital of Tianjin Medical University, Tianjin 300052, China

## Abstract

We investigated the effect of 1.4% isoflurane (ISO) on the development of inflammation and apoptosis caused by zymosan (ZY) in mice. We found that ZY-challenged mice exhibited significant body weight loss, markedly high mortality, and significant lung injury characterized by the deterioration of histopathology, histologic scores, and wet-to-dry ratio after ISO treatment. ISO dramatically attenuated ZY-induced lung neutrophil recruitment and inflammation, as evidenced by the reduced levels of total cells, neutrophils, and proinflammatory cytokines (i.e., tumor necrosis factor-**α**, interleukin- (IL-) 1**β**, IL-6, and macrophage inflammatory protein-2) in bronchoalveolar lavage fluid and of their mRNA expression in lung tissues. ISO also inhibited ZY-induced expression and activation of nuclear factor-kappaB p65 and inducible nitric oxide synthase in pulmonary tissue. ZY administration also resulted in the upregulation of heme oxygenase-1 expression and activity in the lung, which was further enhanced by ISO treatment. Moreover, ISO markedly prevented ZY-induced pulmonary cell apoptosis in mice, as reflected by the decrease in expression of procaspase-8, procaspase-3, cleaved caspase-8, and cleaved caspase-3, as well as in caspase-3 activity and Bcl-2-associated X/B-cell lymphoma 2 ratio. These results indicate that ISO is a potential therapeutic drug for treating ZY-induced lung injury, and further investigations are warranted.

## 1. Introduction

Multiple organ dysfunction syndrome (MODS) is one of the most urgent and challenging public health problems worldwide. In the USA alone, the yearly incidence of MODS is approximately 75 cases per 1 × 10^5^ people, which accounts for 2% of hospital admissions [1, 2]. Lung tissue usually fails first in the development of MODS and intense inflammation, and inflammatory-stress-induced apoptosis is the main cause of lung injury [3–5]. Despite considerable research, the mechanism underlying inflammation-induced lung injury and appropriate treatment approaches are still unknown.

In 1986, Goris et al. [6] described a zymosan- (ZY-) induced generalized inflammation (ZIGI) model, which is recognized as the only model to share numerous characteristics with human MODS and has been adopted by other research groups [6] including our group [7]. ZY is a substance derived from the cell wall of the yeast *Saccharomyces cerevisiae*. When injected into animals, ZY induces inflammation by upregulating a wide range of inflammatory mediators [8–10]. However, intensive inflammation has also been associated with apoptotic cell death [11]. ZY administration also results in the upregulation of proapoptotic Fas ligand and altered balance between Bcl-2-associated X protein (Bax) and B-cell lymphoma 2 (Bcl-2) during inflammation, which contributes to organ injury [12]. Therefore, ZY-induced inflammatory and apoptotic responses are critical contributors to the development of MODS, including lung damage.

Isoflurane (ISO) is a widely used inhaled anesthetic with protective properties ascribed to its anti-inflammatory or antiapoptotic activities [13, 14]. The anti-inflammatory effects of the volatile anesthetic ISO can be explained by several mechanisms, most of which focus on preconditioning [15]. The development of inflammatory lung injury is associated with the activation of complex cytokine cascades. The systemic production of proinflammatory cytokines (tumor necrosis factor-*α* (TNF-*α*), interleukin- (IL-) 1*β*, and IL-6) and nitric oxide (NO) causes inflammatory cell recruitment and activation [16, 17]. Several studies have revealed that using volatile anesthetics reduces the expression of inflammatory mediators and the mRNA expression of proinflammatory cytokines [18]. Nuclear factor- (NF-) *κ*B is a key transcription factor with an important function in inflammatory response. NF-*κ*B activation induces the expression of various inflammation-related products, including cytokines, chemokines, and adhesion molecules [19]. ISO pretreatment can attenuate inflammation in lipopolysaccharide- (LPS-) treated lung by inhibiting NF-*κ*B activation [20]. Neutrophils are an important component of the inflammatory response that characterizes lung injury. Neutrophil recruitment into the lung depends on adhesion molecules and chemokines [21]. In vitro data have suggested that ISO decreases expression of *β*2-integrins in neutrophils stimulated with *N*-formyl-methionyl-leucyl-phenylalanine [22]. Most importantly, anti-inflammatory activity of ISO pretreatment at anesthetic concentrations (1.4%–2.5%) is associated with ameliorated lung dysfunction and mortality [15]. Regarding posttreatment, ISO can reduce neutrophil infiltration into the lung when administered 1 h after an inflammatory stimulus is introduced [15]. Our previous study has shown that ISO post-treatment protects against lung damage caused by ZY because ISO has antioxidant property [7]. However, the protective effects of post-treatment with the volatile anesthetic ISO depending on its anti-inflammation property on ZY-induced inflammatory response in mouse lung remain unknown. ISO pretreatment reportedly inhibits cardiac myocyte apoptosis during inflammatory stress by activating Akt and enhancing Bcl-2 expression [23]. ISO also reduces neonatal hypoxic-ischemic brain injury in rats by decreasing the cleaved caspase-3 levels and protects against human endothelial cell apoptosis caused by TNF-*α* [14, 24]. Similar to ISO pretreatment, ISO post-treatment protects against cell apoptosis triggered by inflammatory stress mainly for cardioprotection and neuroprotection [25–28]. However, in a model of lung injury caused by ZY-induced apoptotic responses, the protective effects of post-treatment with ISO ascribed to its anti-apoptosis property are largely unknown.

Our previous studies only have demonstrated that compared with 0.7% ISO post-treatment, 1.4% ISO post-treatment provides better protection against ZY-induced lung damage given the antioxidant property of ISO [7]. Accordingly, the present study investigated whether 1.4% ISO post-treatment attenuated ZY-induced lung injury in mice through anti-inflammatory and antiapoptotic activities.

## 2. Materials and Methods 

### 2.1. Materials

All reagents were obtained from Sigma-Aldrich (St. Louis, MO, USA) unless otherwise noted. All chemicals were of the highest commercial grade available. ZY A from *S. cerevisiae* was dissolved in isotonic sodium chloride solution (normal saline (NS)) to a final concentration of 25 mg/mL, homogenized by magnetic stirring, and sterilized at 100°C for 80 min. All suspensions were freshly made before use.

### 2.2. Animals

Male BALB/C mice (8 weeks old and weighing 22 g to 25 g) from the Laboratory Animal Center of the Fourth Military Medical University were maintained at a comfortable temperature (22°C to 24°C), regular 12 h day/night cycle, and standard laboratory chow and tap water available *ad libitum.* The Institutional Animal Research Ethics board of the Fourth Military Medical University approved all experimental protocols. Each animal received humane treatment in full compliance with the National Institutes of Health (Bethesda, MD, USA) criteria for the Care and Use of Laboratory Animals. Mice were sacrificed under anesthesia with sodium pentobarbital, and all efforts to minimize suffering were exerted. Euthanasia by sodium pentobarbital was performed in accordance with the American Veterinary Medical Association Guidelines on Euthanasia, June 2007.

### 2.3. ZY-Induced Lung Injury and ISO Treatment

As previously described in [7], a ZY-induced inflammatory lung injury model was established by an aseptic intraperitoneal (IP) injection of ZY in mice with a dose of 1 g/kg body weight (BW). The same volume of NS was injected through the same route in mice to serve as sham controls. 

The animals were placed in a sealed plexiglass chamber with inflow and outflow outlets. ISO was delivered by air flow into the chamber through a tube at a rate of 4 L/min. The ISO concentration in the outflow hose of the chamber was continuously monitored with a gas analyzer (Brüel & Kjaer, Naerum, Denmark) and maintained at 1.4% during treatment. The oxygen concentration in the chamber was maintained at 21% using supplemental oxygen and continuously monitored with a gas analyzer (Medical Gas Analyzer LB-2, Model 40 M; Beckman, Fullerton, CA, USA). Carbon dioxide was removed from the chamber gases with Baralyme (Allied Healthcare Products, Inc., St. Louis, MO, USA). Animals without ISO treatment were exposed to room air (RA) in the chamber as a vehicle control. The temperature of the room and the chamber was maintained at 22°C to 24°C.

### 2.4. Experimental Design

Eighty mice were randomly allocated as follows. (1) ZY + vehicle group: mice were given an IP injection of ZY (1 g/kg, dissolved in NS solution), followed by inhalation of RA (vehicle) for 1 h starting at 1 h and 6 h after ZY administration (*n* = 20). (2) ZY + ISO group: no differences from the ZY + vehicle group, except for 1 h inhalation of 1.4% ISO starting at 1 h and 6 h instead of RA after ZY administration (*n* = 20). (3) Sham + vehicle group: no differences from the ZY + vehicle group, except for administration with NS (Sham) instead of ZY (*n* = 20). (4) Sham + ISO group: identical to the sham + vehicle group, except for 1 h inhalation of 1.4% ISO starting at 1 h and 6 h after NS (sham) administration (*n* = 20). At 24 h after ZY or NS administration, the animals were assessed for ZY-induced lung injury as described below. In another set of experiments, animals (*n* = 20 each group) were randomly assigned as described above and monitored for survival 10 days after ZY or NS administration. 

### 2.5. Wet/Dry Weight Ratio

To quantify pulmonary edema, we evaluated lung wet/dry (W/D) weight ratio 24 h after ZY or NS administration. The harvested wet lung was weighed, placed in an oven for 24 h at 80°C, and weighed again when it dried. The lung W/D ratio was then calculated [29].

### 2.6. Histologic Examination

Lungs were harvested to observe morphological alterations 24 h after ZY or NS administration. The samples were fixed with 10% formalin for 8 h at room temperature, embedded in paraffin, and sectioned to 4 *μ*m thickness. After deparaffinization and rehydration, the sections were stained with hematoxylin and eosin (HE). Histologic changes were evaluated by two independent pathologists who had no knowledge of the treatment regimen received by each respective animal. The degree of lung injury was scored based on a subjective scale ranging from 0 to 3, where 0 = absence, 1 = mild, 2 = moderate, and 3 = severe. The ranging scale was used for each of the following histologic features: edema, hyperemia and congestion, neutrophil margination and tissue infiltration, intra-alveolar hemorrhage and debris, and cellular hyperplasia. The total score is the sum of a single evaluation [30].

### 2.7. Sample Preparation and Cell Counts

At 24 h after ZY or NS administration, bronchoalveolar lavage fluid (BALF) was collected using a previously described method [31]. All animals were anesthetized with sodium pentobarbital (50 mg/kg, IP),tracheas were cannulated after exsanguination, and lungs were gently washed with 1 mL of phosphate-buffered saline (PBS). Lavage samples were centrifuged at 1000 g for 8 min at 4°C. The supernatant was stored at −20°C for subsequent analysis. Cell pellets were resuspended in PBS, and the total number of cells was determined with a hemocytometer (Beckman Coulter, Inc.). For differential cell counts, cytospin slides were prepared and stained with Diff-Quick [32], and each cell type was identified by an independent and certified investigator that had no knowledge of the treatment regimen received by each respective animal.

### 2.8. Enzyme-Linked Immunosorbent Assay

At 24 h after ZY or NS injection, enzyme-linked immunosorbent assay (ELISA) was performed to determine the levels of the pro-inflammation cytokines TNF-*α*, IL-1*β*, IL-6, and macrophage inflammatory protein-2 (MIP-2) using four commercially available kits from R&D Systems Inc. (Minneapolis, MN, USA) in BALF. The optical density was measured on an ELISA plate scanner (Molecular Devices, Sunnyvale, CA, USA) at 490 nm. All experiments were carried out according to the manufacturers' instructions. The results were expressed as picograms of TNF-*α*, IL-1*β*, IL-6, and MIP-2 per milliliter of BALF. All standards and samples were run in duplicate.

### 2.9. Heme Oxygenase-1 (HO-1) Activity Assay

HO-1 activity was quantified by the spectrophotometric determination of bilirubin generation as previously described [33]. At 24 h after ZY or NS injection, the supernatant from homogenated lung tissue was incubated for 1 h in a 37°C water bath in the dark with nicotinamide adenine dinucleotide phosphate (NADPH; 20 mM), hemin (10 mM), and 1 mg of liver cytosol protein (source of bilirubin reductase). After chloroform extraction, bilirubin formation was determined from the absorbance difference at 464 and 530 nm.

### 2.10. Determination of Inducible NO Synthase (iNOS) Activity

iNOS activity was measured by monitoring the conversion of arginine to citrulline based on a previously described standard assay [34]. At 24 h after ZY or NS administration, a 10 *μ*L aliquot of homogenated lung tissue was incubated with L-[^3^H] arginine and the essential substrates and cofactors (tetrahydrobiopterin, NADPH, flavin adenine dinucleotide, etc.). The production of L-[^3^H] citrulline was calculated by liquid scintillation counting. To quantify iNOS activity, ethylenediamine tetraacetic acid (EDTA) and ethylene glycol tetraacetic acid (EGTA) were sequentially added to the incubation buffer. An appropriate blank containing 1 mM l-NG-nitroarginine methyl ester (l-NAME), a competitive iNOS inhibitor, was used to determine the effect from the background of the nonspecific metabolism of l-arginine. iNOS activity in the citrulline assay was determined by the inhibitable degree of l-NAME in the EDTA-EGTA sample, and iNOS expression was expressed in units (where 1 unit = 1 pmol l-citrulline/mg protein/min).

### 2.11. Preparation of Cell Fractions and Western Blot Analysis

At 24 h after ZY or NS injection, cytosolic and nuclear extracts of fresh lung tissues from all groups were prepared by a previous method with some modifications [35]. About 100 mg of lung samples was homogenized in tissue lysis and extraction buffer containing protease inhibitor cocktail set III (EMD Chemicals Inc., Germany). The crude homogenates were repetitively filtered and centrifuged at 15000 ×g for 25 min at 4°C. The supernatants were collected as cytosolic fractions. The nuclei-rich pellets were resuspended in nuclear protein extraction buffer containing 20 mM 4-(2-hydroxyethyl)-1-piperazineethanesulfonic acid (pH 7.9), 10% glycerol, 1 mM dithiothreitol, 400 mM NaCl, 1 mM EDTA, and cocktail set III. After 20 min of centrifugation at 15000 ×g and 4°C, the supernatants containing nuclear protein were pooled. The NF-*κ*B p65 levels were quantified in nuclear fractions. All other protein levels were quantified in cytosolic fractions. The final two extracts (cytosolic and nuclear protein) were boiled, separated by sodium dodecyl sulfate polyacrylamide gel electrophoresis, electrotransferred onto nitrocellulose membranes, and then immunoblotted with rabbit anti-NF-*κ*B polyclonal antibody (pAb), rabbit anti-I*κ*B-*α* pAb, rabbit anti-caspase-3 pAb, and rabbit anti-caspase-8 pAb (Santa Cruz Biotechnology, CA, USA), as well as rabbit anti-HO-1 pAb, rabbit anti-iNOS pAb, rabbit anti-Bax pAb, and rabbit anti-Bcl-2 pAb (Cell Signaling Technology Inc., Danvers, MA, USA). Equivalent sample loading was confirmed by probing with mouse anti-*β*-actin monoclonal antibody (mAb) and rabbit anti-histone-3 mAb (Sigma, CA, USA), respectively. Detection was performed with an enhanced chemiluminescence assay kit (Pierce, Rockford, IL, USA). Protein bands were quantified using Quantity One software (BioRad, USA).

### 2.12. Analysis of mRNA Levels by Reverse Transcription-Polymerase Chain Reaction (RT-PCR)

At 24 h after ZY or NS administration, total RNA was isolated and extracted from the lung tissues from all groups with TRIzol Reagent (Invitrogen, Carlsbad, CA, USA), following the manufacturer's instructions. RT-PCR was performed as previously described [36]. cDNA was first synthesized from total RNA with a SuperScript RT kit (Invitrogen) and then used as a template to amplify TNF-*α*, IL-1*β*, IL-6, MIP-2, HO-1, iNOS, and *β*-actin (as an internal standard) genes by PCR. The following primers were used: TNF-*α*, 5′-TTGACCTCAGC GCTGAGTTG-3′, and 5′-CCTGTAGCCCACGTCGTAGC-3′; IL-1*β*, 5′-CAGGATGAGGACA TGAGCACC-3′ and 5′-CTCTGCAGACTCAAACTCCAC-3′; IL-6, 5′-GTACTCCAGAAGACCAGAGG-3′, and 5′-TGCTGGTGACAACCACGGCC-3′; MIP-2, 5′-CCAAGGGTTGACTTCAAGAAC-3′, and 5′-AGCGAGGCACATCAGGTACG-3′; HO-1, 5′-TCCCAGACACCGCTCCTCC AG-3′, and 5′-GGATTTGGGGCTGCTGGTTTC-3′; iNOS, 5′-CCCTTCCGAAGTTTCTGGCAGCAGC-3′, and 5′-GGCTGTCAGAGCCTCGTGGCTTTGG-3′; and *β*-actin, 5′-GTGGGCCGCCCTAGGC ACCAG-3′, and 5′-GGAGGAAGAGGATGCGGCAGT-3′. The amplified DNA products were separated on 1% agarose gels and photographed using an electronic documentation system (Biostep, Germany) after staining with ethidium bromide. Signal intensities were quantified with Quantity One software (BioRad, USA).

### 2.13. Caspase-3 Activity

At 24 h after ZY or NS administration, lung homogenates were prepared and caspase-3 activity was measured with a caspase-3/CPP32 Fluorometric Assay Kit (Biovision, Inc, Mountain View, CA, USA) according to the manufacturer's instructions [37]. The assay was run in duplicate.

### 2.14. Immunohistochemistry

Immunohistochemistry was performed as previously described [10]. In a typical procedure, 24 h after ZY or NS injection, the lung sections were stained with rabbit anti-Bax mAb and rabbit anti-Bcl-2 mAb (Santa Cruz, CA, USA) at 1 : 200 and 1 : 100 dilutions, respectively. The immunohistochemical slides were blindly observed by two experienced pathologists.

### 2.15. Statistical Analyses

 All data in the text and figures are expressed as mean ± SD. Survival data were calculated using the Fisher exact probability test and are expressed as percentages. An independent, two-tailed Mann-Whitney *U* test was used to compare BW medians. The immunohistochemical results were analyzed by one-way ANOVA followed by Bonferroni's post hoc test for multiple comparisons. Other intergroup differences were tested by one-way ANOVA, followed by a least significant difference *t*-test for multiple comparisons. GraphPad statistical software (GraphPad Software, Inc., San Diego, CA, USA) was used to perform data analysis. In all tests, *P* < 0.05 was considered statistically significant.

## 3. Results

### 3.1. Effect of 1.4% ISO Treatment on ZY-Induced BW Loss, Mortality, and Lung Damage

**Figure 2:**
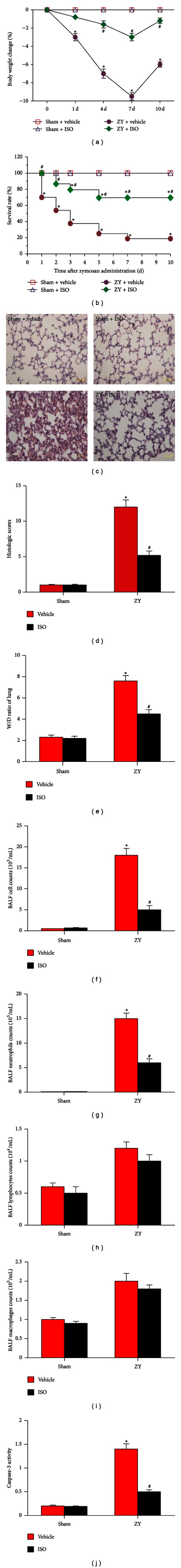
Treatment with 1.4% ISO attenuates lung damage in ZY-challenged mice. (a) Body weight change, (b) survival rate, (c) HE examination of lung (400x magnification), (d) lung histologic scores, (e) lung W/D ratio, (f) BALF total cell counts, (g) BALF neutrophil count, (h) BALF lymphocyte count, (i) BALF macrophage count, and (j) caspase-3 activity. The animals were treated as described in Figure 1. Data are mean ± SD (*n* = 10 mice per group). *P < 0.05 versus sham; ^#^ *P* < 0.05 versus ZY + vehicle.

In this study, we found that ZY administration caused significant BW loss in mice (*P* < 0.05; Figure 2(a)). The 10-day survival rate of ZY-challenged mice was 20% (*P* < 0.05; Figure 2(b)). Treatment with 1.4% ISO significantly reduced loss in BW (*P* < 0.05; Figure 2(a)) and mortality (*P* < 0.05; Figure 2(b)) caused by ZY. However, no significant change in these parameters was observed in sham-treated mice (*P* > 0.05; Figures 2(a) and 2(b)). Moreover, no significant difference was observed between the sham + vehicle and sham + ISO groups. All mice in the sham groups survived throughout the experiment. We also investigated the effects of 1.4% ISO inhalation on lung histopathology in mice with either NS or ZY challenge (Figures 2(c) and 2(d)). ZY-challenged mice appeared to have significant lung injury characterized by alveolar space, consolidation, neutrophil infiltration into lung interstitium, and alveolar wall thickening (Figure 2(c)). HE-stained sections of lungs showed that ISO treatment reduced infiltrated inflammatory cells and improved lung architecture in ZY-challenged mice (Figure 2(c)). A scoring system was used to grade the degree of lung injury. Lung histologic scores significantly increased in ZY-challenged mice (*P* < 0.05; Figure 2(d)) but were reduced by ISO treatment (*P* < 0.05; Figure 2(d)). ZY-challenged mice showed significantly increased lung W/D ratio compared with the sham group, which was also decreased by ISO treatment (*P* < 0.05; Figure 2(e)). At 24 h after IP injection with ZY, total cells and polymorphonuclear neutrophils (PMNs) in BALF significantly increased (*P* < 0.05; Figures 2(f) and 2(g)); however, the number of BALF macrophages and lymphocytes remained almost unchanged (Figures 2(h) and 2(i)). ISO treatment markedly reduced the number of total cells and PMNs in BALF (*P* < 0.05; Figures 2(f) and 2(g)) but had little effect on macrophage and lymphocyte infiltration into the lung (Figures 2(h) and 2(i)) in ZY-challenged mice. No significant alteration was observed in the number of all cell types described above in BALF from sham-treated mice (*P* > 0.05; Figures 2(f)–2(i)). The data indicated no significant difference between the sham + vehicle and sham + ISO groups. We also investigated the effect of ISO treatment on pulmonary cell apoptosis in ZY-challenged mice by assaying caspase-3 activity. Numerous lung cells had caspase-3 activity in ZY-challenged mice (*P* < 0.05; Figure 2(j)). However, a few of the lung cells showed caspase-3 activity in ISO-treated mice (*P* < 0.05; Figure 2(j)). Caspase-3 activity assay showed few apoptotic cells in the lungs from sham-treated mice (*P* > 0.05; Figure 2(j)). No significant difference was observed between the sham + vehicle and sham + ISO groups. These results suggested that ISO treatment significantly alleviated lung injury in ZY-challenged mice.

### 3.2. Effect of 1.4% ISO Treatment on I*κ*B-*α* Degradation and NF-*κ*B p65 Activation

**Figure 3:**
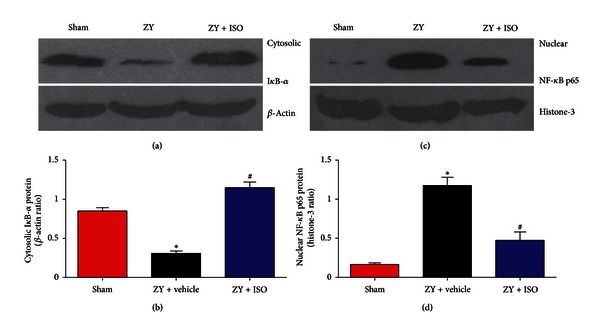
Treatment with 1.4% ISO reduces NF-*κ*B activation caused by ZY in the lung. (a) Western blot analysis of I*κ*B-*α* expression in the cytoplasm, (b) protein bands densitometrically quantified and relative amounts of I*κ*B-*α* protein normalized against *β*-actin, (c) Western blot analysis of the nuclear levels of NF-*κ*B p65, and (d) protein bands densitometrically quantified and relative amounts of NF-*κ*B p65 protein normalized against histone-3. The respective densitometric analysis of protein bands is from three separate experiments. The animals were treated as described in Figure 1. Data are mean ± SD (*n* = 10 mice per group). *P < 0.05 versus sham; ^#^ *P* < 0.05 versus ZY + vehicle.

The cytosolic I*κ*B-*α* and nuclear NF-*κ*B p65 levels were detected to investigate the involvement of NF-*κ*B p65 activation in ZY-induced lung injury. Lung tissue samples from sham-operated mice showed a basal I*κ*B-*α* level, whereas the protein was substantially degraded in ZY-injected mice (*P* < 0.05; Figures 3(a) and 3(b)). However, 1.4% ISO treatment significantly inhibited ZY-induced I*κ*B-*α* degradation in the lung (*P* < 0.05; Figures 3(a) and 3(b)). The nuclear translocation of NF-*κ*B p65 markedly increased in ZY-challenged mice compared with sham-treated mice, which was significantly reduced by 1.4% ISO (*P* < 0.05; Figures 3(c) and 3(d)). The data demonstrate that 1.4% ISO treatment inhibited lung NF-*κ*B activation in ZY-challenged mice.

### 3.3. Effect of 1.4% ISO Treatment on Cytokine Production

**Figure 4:**
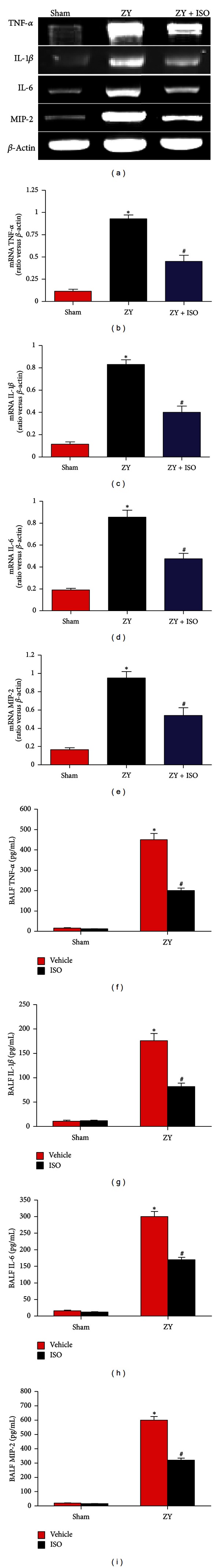
Treatment with 1.4% ISO downregulates proinflammatory cytokine levels in lung tissues and BALF from ZY-challenged mice. (a) RT-PCR analysis for the expression of TNF-*α*, IL-1*β*, IL-6, and MIP-2 mRNA; (b)–(e) amplicons were densitometrically quantified, and bars represent the relative amounts of amplified TNF-*α*, IL-1*β*, IL-6, and MIP-2 mRNA normalized against *β*-actin; (f) BALF TNF-*α*; (g) BALF IL-1*β*; (h) BALF IL-6; (i) BALF MIP-2. BALF was obtained to measure these indicators using ELISA kits. The respective densitometric analysis of mRNA bands is from three separate experiments. The animals were treated as described in Figure 1. Data are mean ± SD (*n* = 10 mice per group). *P < 0.05 versus sham; ^#^ *P* < 0.05 versus ZY + vehicle.

As shown in Figure 4, ZY-challenged mice exhibited a markedly increased mRNA expression of proinflammatory cytokines (TNF-*α*, IL-1*β*, IL-6, and MIP-2) in lung tissue. Treatment with 1.4% ISO significantly inhibited the expression of the aforementioned molecules. We also found that the levels of the proinflammatory cytokines TNF-*α*, IL-1*β*, IL-6, and MIP-2 in BALF substantially increased at 24 h in ZY-challenged mice, which were markedly downregulated by 1.4% ISO treatment. The BALF and pulmonary levels of these cytokines were almost not altered in sham-treated mice. These results demonstrated that 1.4% ISO treatment significantly prevented proinflammatory cytokine (TNF-*α*, IL-1*β*, IL-6, and MIP-2) upregulation in BALF and lung tissues of ZY-challenged mice.

### 3.4. Effect of 1.4% ISO Treatment on the Expression and Activation of HO-1 and iNOS

**Figure 5:**
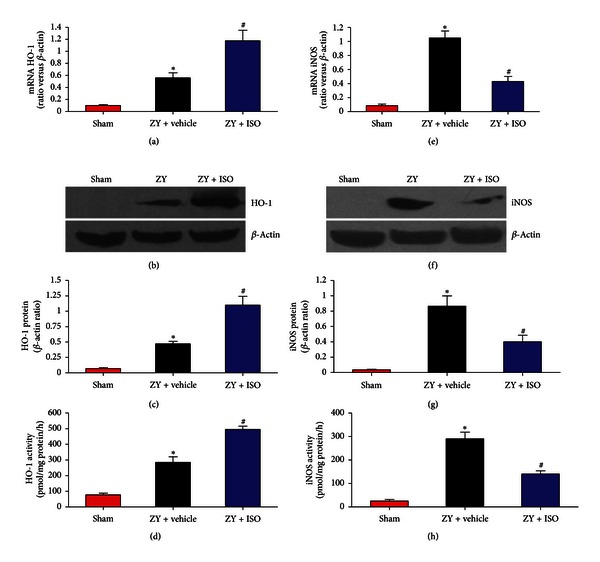
HO-1 activation and iNOS suppression induced by 1.4% ISO treatment in ZY-stimulated lungs. Amplicons were densitometrically quantified, and the relative amounts of amplified HO-1 mRNA (a) and iNOS mRNA (e) were normalized against *β*-actin; Western blot analysis for expression of HO-1 (b) and iNOS (f); protein bands were densitometrically quantified and relative amounts of HO-1 protein (c) and iNOS protein (g) were normalized against *β*-actin; and detection of HO-1 (d) and iNOS (h) enzyme activities. The respective densitometric analysis of mRNA and protein bands is from three separate experiments. The animals were treated as described in Figure 1. Data are mean ± SD (*n* = 10 mice per group). *P < 0.05 versus sham; ^#^ *P* < 0.05 versus ZY + vehicle.

We evaluated the expression and activities of HO-1 and iNOS to investigate the protective mechanisms of 1.4% ISO treatment. The mRNA and protein levels of HO-1 and its activity significantly increased in mouse lungs 24 h after ZY administration compared with the sham control (*P* < 0.05; Figures 5(a)–5(d)). Treatment with 1.4% ISO further increased the expression and activities of HO-1 (*P* < 0.05; Figures 5(a)–5(d)). The mRNA and protein levels of iNOS and its activity also significantly increased in mouse lungs 24 h after ZY challenge compared with the sham control but were significantly reduced after 1.4% ISO treatment (*P* < 0.05; Figures 5(e)–5(h)). Thus, 1.4% ISO upregulated HO-1 expression and activity but downregulated iNOS expression and activity.

### 3.5. Effect of 1.4% ISO Treatment on Apoptosis Initiated by ZY Stimulation

**Figure 6:**
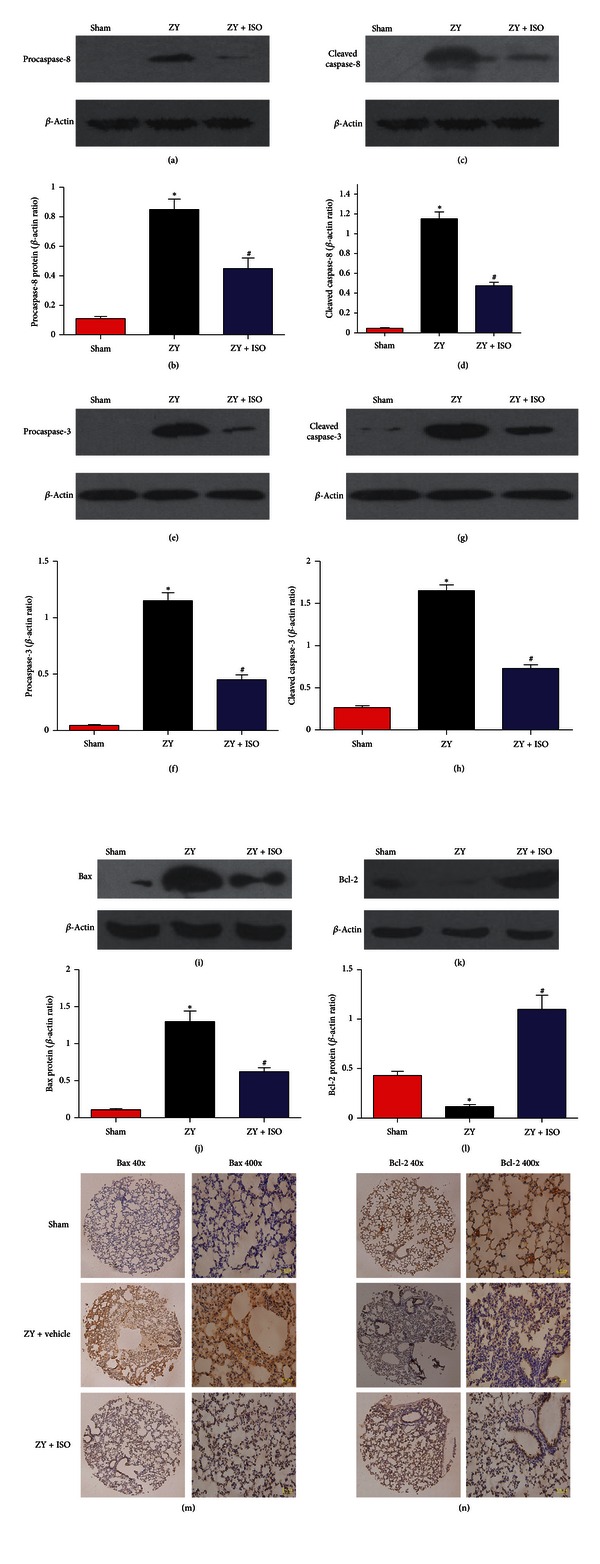
Treatment with 1.4% ISO inhibits pulmonary cell apoptosis in ZY-challenged mice. Western blot analysis for expression of procaspase-8 (a), cleaved caspase-8 (c), procaspase-3 (e), cleaved caspase-3 (g), Bax (i), and Bcl-2 (k); protein bands were densitometrically quantified and relative amounts of procaspase-8 (b), cleaved caspase-8 (d), procaspase-3 (f), cleaved caspase-3 (h), Bax (j), and Bcl-2 (l) are shown; histologic sections were stained with anti-Bax antibody ((m); 40x or 400x magnification) and anti-Bcl-2 antibody ((n); 40x or 400x magnification). The respective densitometric analysis of protein bands is from three separate experiments. Immunohistochemistry figures are representative of at least three experiments performed on different experimental days. The animals were treated as described in Figure 1. Data are mean ± SD (*n* = 10 mice per group). *P < 0.05 versus sham; ^#^ *P* < 0.05 versus ZY + vehicle.

Inflammatory stress usually causes lung cell apoptosis. As shown in Figure 2(j), 1.4% ISO treatment inhibited pulmonary cell apoptosis in ZY-challenged mice by downregulating caspase-3 activity. We further identified other molecules that may be involved in ZY-induced lung cell apoptosis as well as the effect of 1.4% ISO treatment on their expression and activity. As shown in Figure 6, ZY injection dramatically promoted the expression of proapoptotic molecules (procaspase-8, cleaved caspase-8, procaspase-3, cleaved caspase-3, and Bax) in the lung, which were significantly prevented by 1.4% ISO treatment. However, ZY administration markedly hindered the protein expression of the antiapoptotic Bcl-2 in the lung. Treatment with 1.4% ISO significantly reversed this inhibition. In addition, the expression of proapoptotic and antiapoptotic molecules was almost unchanged in lung tissues from sham-treated mice. The expression changes of Bax and Bcl-2 in different groups according to the immunohistochemical observations of the two experienced pathologists were similar to those of the Western blot assay results. These results demonstrated that 1.4% ISO treatment attenuated ZY-induced lung injury by modulating the expression of proapoptotic and antiapoptotic molecules.

## 4. Discussion

An animal model of IP injection of ZY enables a systematic investigation of the development and mechanisms of lung injury. Such a model can help understand the pathophysiology and treatment of ZY-induced inflammation and apoptosis. ISO is a widely used inhaled anesthetic with protective properties ascribed to its antioxidant, anti-inflammatory, or antiapoptotic activities [14, 15, 31]. Our previous studies have demonstrated that 1.4% ISO inhalation after ZY injection confers protection against ZY-induced lung injury and lethal shock in mice. This protective effect mainly stems from the antioxidant properties of ISO [7]. However, the anti-inflammatory and antiapoptotic effects of ISO post-treatment in ZY-induced lung injury still await exploration. In the present study, a series of experiments was performed to understand further the function of 1.4% ISO during ZY-induced lung damage. The notable findings of our study were as follows: (1) ISO prevented BW loss and improved the survival rate of ZY-challenged mice; (2) ISO reduced the lung histologic scores and decreased the lung W/D ratio in ZY-challenged mice; (3) ISO reduced the total number of cells in BALF mainly by inhibiting neutrophil infiltration; (4) ISO inhibited proinflammatory cytokine production in lung tissue and BALF; (5) ISO reduced NF-*κ*B p65 expression and blocked its activation; (6) ISO lowered iNOS expression and its activity; (7) ISO enhanced HO-1 expression and its activity; and (8) ISO suppressed apoptosis by downregulating the expression of procaspase-8, caspase-8, procaspase-3, and caspase-3, as well as by modulating Bax and Bcl-2 expression and ratio. Therefore, 1.4% ISO significantly reduced the degree of ZY-induced lung injury in mice by decreasing ongoing inflammation and inhibiting apoptosis.

ZY is prepared from *S. cerevisiae* and is recognized by Toll-like receptor-2 in immune cells (e.g., neutrophils), which can trigger a signal cascade for NF-*κ*B activation. NF-*κ*B activation is a critical transcription factor required for the maximal expression of many inflammatory cytokines and chemokines (e.g., TNF-*α*, IL-1*β*, IL-6, and MIP-2) involved in the pathogenesis of acute lung injury [38]. The excessive production of inflammatory mediators such as TNF-*α*, IL-1*β*, and IL-6 propagated the extension of inflammation and facilitated systemic inflammation, ultimately contributing to the overall outcome and severity [39]. MIP-2 is a major chemokine with a key function in the infiltration of neutrophils into the lung. Pulmonary neutrophil accumulation and activation is the initial event of tissue injury and can indicate the magnitude of disease severity [38]. In our mouse model of ZY-induced lung injury, we observed that two times of 1.4% ISO exposure for 1 h significantly inhibited NF-*κ*B p65 expression and blocked its activation. NF-*κ*B p65 activation promoted TNF-*α* and IL-1*β* transcription. Accordingly, 1.4% ISO treatment conferred considerable protection through the promotion of the effective remission of inflammatory responses by suppressing the expression of the proinflammatory cytokines TNF-*α*, IL-1*β*, IL-6, and MIP-2 in lung tissue and BALF. The beneficial effect of ISO treatment in ZY-induced lung damage appeared to be mediated by the inhibition of NF-*κ*B activation. The high levels of TNF-*α*, IL-1*β*, and MIP-2 also caused neutrophilic inflammation and decreased pulmonary function, which depended on the recruitment of neutrophils from the vascular space to the airspace [10, 38]. Our study indicated that 1.4% ISO exposure significantly reduced the number of total cells mainly by inhibiting neutrophil infiltration into the lung 24 h after ZY injection. These data demonstrated that 1.4% ISO exerted functional protective effects in the lung following ZY challenge, which depended on the anti-inflammatory activity of ISO.

Inflammatory activity is mediated by the endogenous free radical NO, which is produced at high levels upon the induction of NO synthase by inflammatory stimulus [40]. In an experimental murine model, ZY administration increases iNOS expression and activity that exacerbates nonseptic shock and leads to cellular and tissue damage including lung injury if unchecked. However, iNOS inhibitors suppress airway inflammation in mice by downregulating proinflammation and chemokine expression that are detrimental to the lung. iNOS-deficient mice also undergo less lung injury after ZY challenge [41]. In the present study, we demonstrated that ISO treatment caused several molecular lesions in iNOS expression and its activity, and this phenomenon significantly inhibited the inflammatory response. Moreover, NF-*κ*B is a potent regulator of iNOS expression [42], and the aforementioned results paralleled iNOS expression and its activity as assessed by activating NF-*κ*B [43]. Based on these findings, we speculated that ISO probably inhibited iNOS expression and activity by blocking NF-*κ*B activation. However, further studies are needed to verify this speculation.

The stress response protein HO-1 with three products (carbon monoxide (CO), iron, and biliverdin) has been demonstrated by numerous laboratories to offer protection against inflammation and apoptosis in models of shock including lung injury in mice. HO-1 exerts its protective effects through biliverdin or CO. CO possesses anti-inflammatory properties by regulating various cytokine expressions (e.g., TNF-*α*, IL-1*β*, and IL-6) following LPS challenge [44]. CO also protects many cells from inflammation stress through antiapoptotic mechanisms [45]. In the present study, enzyme activity assessment and Western blot analyses of lung tissues showed that ISO treatment augmented HO-1 expression and activity. Thus, increased HO-1 expression and activity in the lung possibly acted as a server to provide endogenous CO, which had potent cytoprotective (e.g., antiapoptotic) properties. Zhang et al. [46] reported that HO-1 overexpression in mouse lung increases Bcl-2 expression but decreases Bax expression. In the current work, ISO treatment induced the mRNA and protein expression of HO-1 and its activity. Under the same conditions, Bcl-2 expression paralleled HO-1 expression and its activity, whereas Bax expression showed the opposite tendency. Based on the aforementioned data, we speculated that HO-1 upregulation caused by ISO possibly promoted Bcl-2 expression but suppressed Bax expression, although this speculation requires verification. 

Proinflammatory cytokines, free radicals, and iNOS-derived NO have also been associated with apoptotic cell death [11]. Induction of apoptosis has been implicated in the pathophysiology of MODS [47] and may lead to immunological dysfunctions. Therefore, preventing or diminishing apoptosis levels can protect from organ damage. In the current study, we demonstrated that 1.4% ISO treatment attenuated the degree of apoptosis, measured by caspase-3 activity in the lung after ZY administration. At the molecular level, we found that 1.4% ISO inhalation markedly inhibited the upregulation of the proapoptotic procaspase-8, procaspase-3, cleaved caspase-8, cleaved caspase-3, and Bax and promoted the expression of the antiapoptotic Bcl-2 expression using Western blot assay. Immunohistochemical staining also confirmed the expression changes of Bax and Bcl-2. However, the antiapoptotic effect observed after ISO treatment possibly depended partially on the attenuation of the inflammation-induced damage. The prevention of neutrophil apoptosis possibly aggravated and prolonged lung injury rather than promoted resolution. However, Chiang et al. [13] reported that the volatile anesthetic ISO affected neutrophil resolution program(s) and enhanced the timely resolution of acute inflammation in a ZIGI mouse model, thereby altering specific resolution indices and selective cellular/molecular components in inflammation resolution.

The last but most essential aspect of our study was the clinical implication of 1.4% ISO, a potential anti-inflammation and antiapoptotic agent with unique properties. Unlike most known anti-inflammation and antiapoptotic agents, ISO can permeate cell membranes and successfully target organelles, including the cytosol, mitochondria, and nuclei. However, ZY-induced lung injury in mice only partially mimicked the clinical manifestation of human sepsis. Therefore, further investigations are urgently needed to characterize the actions of 1.4% ISO at the cellular and molecular levels, as well as to explore the pharmacological effects of ISO in the clinical setting.

## 5. Conclusions

This study demonstrated that 1.4% ISO post-treatment attenuated ZY-induced lung injury in mice because ISO has anti-inflammatory and antiapoptotic properties. First, ISO reduced lung histologic scores and decreased lung W/D ratio in ZY-challenged mice. Second, ISO inhibited neutrophil infiltration into the lung and reduced proinflammatory cytokine production in ZY-treated lungs. Third, ISO reduced the expression of the proinflammatory signaling molecules NF-*κ*B p65 and iNOS and their activities. Fourth, ISO promoted the expression and activity of the anti-inflammatory and antiapoptotic HO-1. Fifth, 1.4% ISO prevented ZY-induced pulmonary cell apoptosis in mice by suppressing procaspase-8, procaspase-3, cleaved caspase-8, cleaved caspase-3, and Bax expression; reducing caspase-3 activity; promoting Bcl-2 expression. Finally, ISO prevented BW loss and improved the survival rate in ZY-challenged mice. This new knowledge on the anti-inflammation and anti-apoptosis effects of 1.4% ISO against ZY-induced shock can serve as a foundation for the development of new therapeutics. Our results offer new avenues for continued studies on cellular and molecular markers for use in animal models of ZY-induced lung injury, as well as for future translational and clinical research.

## Figures and Tables

**Figure 1:**
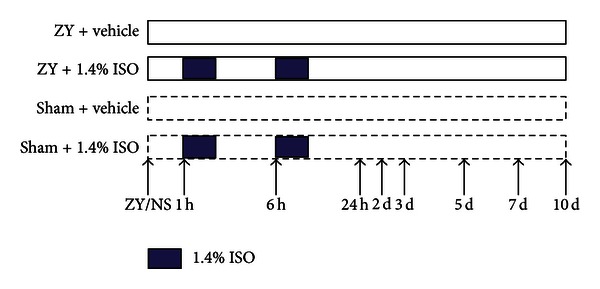
Schematic diagram of the experimental design. The animals were randomly divided into four groups (*n* = 20 per group): ZY + vehicle, ZY + 1.4% ISO, sham + vehicle, and sham + 1.4% ISO. ISO treatment was performed in the ZY + 1.4% ISO and sham + 1.4% ISO groups, with the animals exposed to 1.4% ISO for 1 h starting at 1 and 6 h after ZY or NS injection, respectively. To serve as controls, animals in the sham + vehicle and ZY + vehicle groups were exposed to RA at the same time points. About 24 h after ZY or NS injection, ZY-induced lung damage was observed and assessed in mice. The survival rate was monitored 1, 2, 3, 5, 7, and 10 days after ZY or NS administration.

## Abbreviations

BALF: Bronchoalveolar lavage fluid
ELISA: Enzyme-linked immunosorbent assay
HO-1: Heme oxygenase-1
IL-1*β*: Interleukin-1*β*
iNOS: Inducible nitric oxide synthase
IP: Intraperitoneal
ISO: Isoflurane
MIP-2: Macrophage inflammatory protein-2
MODS: Multiple-organ dysfunction syndrome
NF-*κ*B: Nuclear factor-*κ*B
NS: Normal saline
PBS: Phosphate-buffered saline
PMNs: Polymorphonuclear neutrophils
RA: Room air
TNF-*α*: Tumor necrosis factor-*α*
ZIGI: Zymosan-induced generalized inflammation
ZY: Zymosan.

## References

[1] Bone RC, Sprung CL, Sibbald WJ (1992). Definitions for sepsis and organ failure. *Critical Care Medicine*.

[2] Martin GS, Mannino DM, Eaton S, Moss M (2003). The epidemiology of sepsis in the United States from 1979 through 2000. *New England Journal of Medicine*.

[3] Pugin J, Verghese G, Widmer MC, Matthay MA (1999). The alveolar space is the site of intense inflammatory and profibrotic reactions in the early phase of acute respiratory distress syndrome. *Critical Care Medicine*.

[4] Albertine KH, Soulier MF, Wang Z (2002). Fas and fas ligand are up-regulated in pulmonary edema fluid and lung tissue of patients with acute lung injury and the acute respiratory distress syndrome. *American Journal of Pathology*.

[5] Neff TA, Guo RF, Neff SB (2005). Relationship of acute lung inflammatory injury to Fas/FasL system. *American Journal of Pathology*.

[6] Goris RJA, Boekholtz WKF, Van Bebber IPT (1986). Multiple-organ failure and sepsis without bacteria: an experimental model. *Archives of Surgery*.

[7] Mu J, Xie K, Hou L (2010). Subanesthetic dose of isoflurane protects against zymosan-induced generalized inflammation and its associated acute lung injury in mice. *Shock*.

[8] Hou L, Xie K, Li N (2009). 100% oxygen inhalation protects against zymosan-induced sterile sepsis in mice: the roles of inflammatory cytokines and antioxidant enzymes. *Shock*.

[9] von Asmuth EJU, Maessen JG, Van Der Linden CJ, Buurman WA (1990). Tumour necrosis factor alpha (TNF-*α*) and interleukin 6 in a zymosan-induced shock model. *Scandinavian Journal of Immunology*.

[10] Di Paola R, Mazzon E, Genovese T (2009). Ethyl pyruvate reduces the development of zymosan-induced generalized inflammation in mice. *Critical Care Medicine*.

[11] Washo-Stultz D, Hoglen N, Bernstein H, Bernstein C, Payne CM (1999). Role of nitric oxide and peroxynitrite in bile salt-induced apoptosis: relevance to colon carcinogenesis. *Nutrition and Cancer*.

[12] Di Paola R, Galuppo M, Mazzon E, Paterniti I, Bramanti P, Cuzzocrea S (2010). PD98059, a specific MAP kinase inhibitor, attenuates multiple organ dysfunction syndrome/failure (MODS) induced by zymosan in mice. *Pharmacological Research*.

[13] Chiang N, Schwab JM, Fredman G, Kasuga K, Gelman S, Serhan CN (2008). Anesthetics impact the resolution of inflammation. *PLoS ONE*.

[14] Zhou Y, Lekic T, Fathali N (2010). Isoflurane posttreatment reduces neonatal hypoxic-ischemic brain injury in rats by the sphingosine-1-phosphate/phosphatidylinositol-3-kinase/akt pathway. *Stroke*.

[15] Li QF, Zhu YS, Jiang H, Xu H, Sun Y (2009). Isoflurane preconditioning ameliorates endotoxin-induced acute lung injury and mortality in rats. *Anesthesia and Analgesia*.

[16] Macdonald J, Galley HF, Webster NR (2003). Oxidative stress and gene expression in sepsis. *British Journal of Anaesthesia*.

[17] Kabir K, Gelinas JP, Chen M (2002). Characterization of a murine model of endotoxin-induced acute lung injury. *Shock*.

[18] Biao Z, Zhanggang X, Hao J, Changhong M, Jing C (2005). The in vitro effect of desflurane preconditioning on endothelial adhesion molecules and mRNA expression. *Anesthesia and Analgesia*.

[19] Yoshidome H, Kato A, Edwards MJ, Lentsch AB (1999). Interleukin-10 inhibits pulmonary NF-*κ*B activation and lung injury induced by hepatic ischemia-reperfusion. *American Journal of Physiology*.

[20] de Rossi LW, Brueckmann M, Rex S, Barderschneider M, Buhre W, Rossaint R (2004). Xenon and isoflurane differentially modulate lipopolysaccharide-
induced activation of the nuclear transcription factor *κ*B and production of tumor necrosis factor-alpha and interleukin-6 in monocytes. *Anesthesia and Analgesia*.

[21] Kubo H, Doyle NA, Graham L, Bhagwan SD, Quinlan WM, Doerschuk CM (1999). L- and P-selectin and CD11/CD18 in intracapillary neutrophil sequestration in rabbit lungs. *American Journal of Respiratory and Critical Care Medicine*.

[22] de Rossi LW, Horn NA, Buhre W, Gass F, Hutschenreuter G, Rossaint R (2002). The effect of isoflurane on neutrophil selectin and *β*2-integrin activation in vitro. *Anesthesia and Analgesia*.

[23] Jamnicki-Abegg M, Weihrauch D, Pagel PS (2005). Isoflurane inhibits cardiac myocyte apoptosis during oxidative and inflammatory stress by activating Akt and enhancing Bcl-2 expression. *Anesthesiology*.

[24] Bakar AM, Park SW, Kim M, Lee HT (2012). Isoflurane protects against human endothelial cell apoptosis by inducing sphingosine kinase-1 via ERK MAPK. *International Journal of Molecular Sciences*.

[25] Chiari PC, Bienengraeber MW, Pagel PS, Krolikowski JG, Kersten JR, Warltier DC (2005). Isoflurane protects against myocardial infarction during early reperfusion by activation of phosphatidylinositol-3-kinase signal transduction: evidence for anesthetic-induced postconditioning in rabbits. *Anesthesiology*.

[26] Pravdic D, Mio Y, Sedlic F (2010). Isoflurane protects cardiomyocytes and mitochondria by immediate and cytosol-independent action at reperfusion. *British Journal of Pharmacology*.

[27] McMurtrey RJ, Zuo Z (2010). Isoflurane preconditioning and postconditioning in rat hippocampal neurons. *Brain Research*.

[28] Lee JJ, Li A, Jung HH, Zuo Z (2008). Postconditioning with isoflurane reduced ischemia-induced brain injury in rats. *Anesthesiology*.

[29] Xie K, Yu Y, Pei Y (2010). Protective effects of hydrogen gas on murine polymicrobial sepsis via reducing oxidative stress and HMGB1 release. *Shock*.

[30] Lin TN, He YY, Wu G, Khan M, Hsu CY, Marmarou A (1993). Effect of brain edema on infarct volume in a focal cerebral ischemia model in rats. *Stroke*.

[31] Wunschel D, Webb-Robertson BJ, Frevert CW (2009). Differentiation of gram-negative bacterial aerosol exposure using detected markers in bronchial-alveolar lavage fluid. *PLoS ONE*.

[32] Lee YJ, Han JY, Byun J (2012). Inhibiting Mer receptor tyrosine kinase suppresses STAT1, SOCS1/3, and NF-kappaB activation and enhances inflammatory responses in lipopolysaccharide-induced acute lung injury. *Journal of Leukocyte Biology*.

[33] Lam CW, Getting SJ, Perretti M (2005). In vitro and in vivo induction of heme oxygenase 1 in mouse macrophages following melanocortin receptor activation. *Journal of Immunology*.

[34] Cernadas MR, Sánchez de Miguel L, García-Durán M (1998). Expression of constitutive and inducible nitric oxide synthases in the vascular wall of young and aging rats. *Circulation Research*.

[35] Bethea JR, Castro M, Keane RW, Lee TT, Dietrich WD, Yezierski RP (1998). Traumatic spinal cord injury induces nuclear factor-*κ*B activation. *Journal of Neuroscience*.

[36] Xin ZF, Shen CC, Tao LJ, Yan SG, Wu HB (2013). Gambogic acid inhibits invasion of osteosarcoma via upregulation of TIMP-1. *International Journal of Molecular Medicine*.

[37] Huang Y, Xie K, Li J (2011). Beneficial effects of hydrogen gas against spinal cord ischemia-reperfusion injury in rabbits. *Brain Research*.

[38] Blackwell TS, Blackwell TR, Christman JW (1997). Impaired activation of nuclear factor-*κ*B in endotoxin-tolerant rats is associated with down-regulation of chemokine gene expression and inhibition of neutrophilic lung inflammation. *Journal of Immunology*.

[39] Gogos CA, Drosou E, Bassaris HP, Skoutelis A (2000). Pro- versus anti-inflammatory cytokine profile in patients with severe sepsis: a marker for prognosis and future therapeutic options. *Journal of Infectious Diseases*.

[40] Paola RD, Mazzon E, Muià C (2007). Protective effect of Hypericum perforatum in zymosan-induced multiple organ dysfunction syndrome: relationship to its inhibitory effect on nitric oxide production and its peroxynitrite scavenging activity. *Nitric Oxide*.

[41] Cuzzocrea S, Mazzon E, Dugo L (2001). Inducible nitric oxide synthase knockout mice exhibit resistance to the multiple organ failure induced by zymosan. *Shock*.

[42] Xie QW, Kashiwabara Y, Nathan C (1994). Role of transcription factor NF-*κ*B/Rel in induction of nitric oxide synthase. *Journal of Biological Chemistry*.

[43] Kim BH, Hong SS, Kwon SW (2008). Diarctigenin, a lignan constituent from Arctium lappa, down-regulated zymosan-induced transcription of inflammatory genes through suppression of DNA binding ability of nuclear factor-*κ*B in macrophages. *Journal of Pharmacology and Experimental Therapeutics*.

[44] Vicente AM, Guillén MI, Alcaraz MJ (2003). Participation of heme oxygenase-1 in a model of acute inflammation. *Experimental Biology and Medicine*.

[45] Brouard S, Berberat PO, Tobiasch E, Seldon MP, Bach FH, Soares MP (2002). Heme oxygenase-1-derived carbon monoxide requires the activation of transcription factor NF-*κ*B to protect endothelial cells from tumor necrosis factor-*α*-mediated apoptosis. *Journal of Biological Chemistry*.

[46] Zhang X, Shan P, Jiang G (2006). Endothelial STAT3 is essential for the protective effects of HO-1 in oxidant-induced lung injury. *The FASEB Journal*.

[47] Ayala A, Perl M, Venet F, Lomas-Neira J, Swan R, Chung CS (2008). Apoptosis in sepsis: mechanisms, clinical impact and potential therapeutic targets. *Current Pharmaceutical Design*.

